# Integrative multi-omics and machine learning analysis identifies candidate biomarkers associated with mitochondrial quality control in major depressive disorder

**DOI:** 10.3389/fpsyt.2026.1828806

**Published:** 2026-08-12

**Authors:** Jingchun Li, Min Wang, Haoqi Liu, Kaiqiang Dong, Rongjuan Guo

**Affiliations:** 1 Department of Neurology, Dongfang Hospital, Beijing University of Chinese Medicine, Beijing, China; 2 Department of Psychiatry and Psychology, The Third Affiliated Hospital of Southern Medical University, Guangzhou, Guangdong, China

**Keywords:** DCHS1, HS3ST2, machine learning, major depressive disorder, mitochondrial quality control, multi-omics

## Abstract

**Background:**

Major depressive disorder (MDD) possesses a complex pathogenesis, with abnormal mitochondrial quality control (MQC) proposed as a potential mechanism involved in the pathological process.

**Methods:**

This study integrated two microarray expression profiling datasets with a single-nucleus RNA sequencing (snRNA-seq) dataset from the human prefrontal cortex (PFC). Candidate genes were identified by intersecting differentially expressed genes (DEGs) from the training set with MQC-associated module genes identified through WGCNA. Ten machine learning algorithms ranked MQC-associated candidate biomarkers, followed by preliminary mRNA-level verification using PFC tissues from chronic restraint stress (CRS) rats. Additionally, MQC-related gene set activity was computationally inferred at the single-cell level to examine cell-type-specific transcriptional alterations associated with MDD.

**Results:**

The application of ten machine learning algorithms highlighted *DCHS1* and *HS3ST2* as candidate biomarkers linked to MQC-related transcriptional alterations. Gene set enrichment analysis (GSEA) indicated associations of these genes with oxidative phosphorylation and cytokine-cytokine receptor interaction pathways. In CRS rats, *DCHS1* mRNA expression decreased, while *HS3ST2* mRNA expression increased, aligning with bioinformatic findings. Among the 18 annotated cell types in the snRNA-seq dataset, computationally inferred MQRG activity significantly decreased in eight cell types, including several excitatory and inhibitory neuronal subtypes. Inhib_*GRIK1* neurons exhibited cell-type-specific expression differences in *DCHS1* and *HS3ST2*.

**Conclusion:**

*DCHS1* and *HS3ST2* may serve as candidate biomarkers associated with MQC-related transcriptional alterations in the PFC of patients with MDD. MQRG activity demonstrated marked cell-type heterogeneity and reduction across multiple PFC cell populations. These findings provide preliminary, hypothesis-generating evidence for the link between MQC-related transcriptional dysregulation and MDD; however, further functional experiments are necessary to ascertain whether *DCHS1* and *HS3ST2* directly regulate mitochondrial quality control.

## 1 Introduction

Major depressive disorder (MDD) is a common psychiatric condition characterized by persistent low mood and diminished interest or pleasure lasting at least two weeks (1, 2). It ranks among the leading global causes of disability. The pathogenesis of MDD is intricate, involving neurotransmitter imbalances, such as dysregulation of serotonin and norepinephrine, neuroendocrine abnormalities like hyperactivity of the hypothalamic-pituitary-adrenal axis, impaired neuroplasticity, immune-inflammatory responses, dysfunction in mitochondrial energy metabolism, and dysbiosis of gut microbiota. These factors display significant variability among individuals (3, 4).

Several brain regions are implicated in MDD, including the prefrontal cortex (PFC), anterior cingulate gyrus, hippocampus, amygdala, nucleus accumbens, and anterior insular cortex (5–9). A previous study utilizing proton magnetic resonance spectroscopy (1H-MRS) reported decreased GABA concentrations and increased glutamine levels in the PFC of MDD patients (10). Clinical practice indicates that treatments targeting the dorsolateral PFC (dlPFC) can improve depressive symptoms (11). Furthermore, alterations in 1, 2-diacylglycerol (DG) levels and structure have been noted in the PFC of depression-like primates (12). A deficiency of pleiotropic protein within astrocytes of the PFC has also resulted in stress-induced depression-like responses in male mice (13). These findings emphasize the PFC’s critical role in MDD, although the specific molecular mechanisms underlying its contribution to the disorder remain unclear.

Mitochondrial Quality Control (MQC) constitutes a dynamic regulatory network through which cells preserve mitochondrial structural integrity and functional homeostasis. This is primarily accomplished through mechanisms such as mitochondrial biogenesis, fusion and fission, mitophagy, and proteostasis regulation (14, 15). As the central hub of cellular energy metabolism, mitochondrial dysfunction can result in insufficient energy supply, imbalanced oxidative stress, and aberrant apoptosis. MQC is essential for maintaining cellular physiological functions, neuronal survival, and synaptic plasticity by facilitating the timely repair or removal of damaged mitochondria (16, 17). Recent research has made strides in understanding the association between MQC and depression. Notably, mitochondrial respiration and energy deficits show significant negative correlations with depression severity, which encompasses symptoms like impaired concentration and fatigue (18). Investigations have revealed disordered mitochondrial morphology, reduced energy metabolism efficiency, and elevated oxidative stress levels in key brain regions of individuals with depression (19, 20). Previous studies indicate that abnormal expression of the mitochondrial fusion protein MFN2 may contribute to depressive-like behaviors by affecting synaptic function (21). Chronic stress has been shown to promote mitochondrial fission in the medial PFC by activating dynein-related protein 1 (DRP1), leading to depression-like behavior and mitochondrial dysfunction in male mice (22). While the expression of MQC genes in the PFC may be statistically correlated with MDD development, further exploration of candidate biomarkers and molecular mechanisms is essential.

Existing literature highlights the vital role of MQC in the onset and progression of MDD. However, understanding how MQC modulates these processes and identifying its key regulatory genes remains a significant challenge. Technological advancements have enabled the investigation of gene expression profiles in the brain tissue of individuals with depression through transcriptome sequencing. Notably, snRNA-seq technology provides single-cell resolution, allowing for the examination of gene expression heterogeneity across various cell subtypes and contributing a foundational dataset for uncovering cell-type-specific mechanisms (23, 24). The integrated use of these sequencing technologies will enhance the exploration of potential pathological mechanisms and therapeutic targets for depression.

In this study, data from two microarray expression profiling datasets and a snRNA-seq dataset were employed to analyze the pathogenesis of MDD comprehensively. The goal was to identify new targets and strategies for treating this disorder. By applying ten machine learning algorithms, multi-dimensional data mining was conducted to identify key MQRGs in MDD. This approach facilitated the identification of biomarkers and gene modules associated with MDD, providing new insights into the mechanisms underlying the disorder.

## 2 Materials and methods

### 2.1 Source of data

The Gene Expression Omnibus (GEO) database (http://www.ncbi.nlm.nih.gov/geo/) provided datasets related to MDD, specifically GSE92538, GSE54570, and GSE144136. The GSE92538 dataset, serving as the training set and utilizing platform GPL17027, included 47 samples from the dlPFC of MDD patients and 119 control samples (25). The GSE54570 dataset was used as a validation set, based on platform GPL96, and contained samples from the dlPFC, comprising 13 samples from MDD patients and 13 control samples (25). Meanwhile, the GSE144136 dataset, which consisted of single-nucleus RNA-seq data obtained from the GPL20301 sequencing platform, included 17 dlPFC samples from MDD patients and 17 control samples (26). Based on prior research (27), 20 MQRGs were retrieved. These genes primarily participate in three essential processes: mitochondrial biosynthesis and metabolic regulation (e.g., *PPARGC1A*, *PPARA*, *PPARG*, *NRF1*, *NFE2L2*, *TFAM*, *ESRRA*); mitochondrial fusion (e.g., *MFN1*, M*F*N2, *OPA1*); and fission (e.g., *MFF*, *FIS1*, *MIEF1*, *MIEF2*), as well as mitochondrial autophagy (e.g., *PINK1*, *PARK2*, *SQSTM1*) and autophagy markers (e.g., *MAP1LC3A*, *MAP1LC3B*, *MAP1LC3C*).

### 2.2 Differential gene expression identification

Differential expression analysis between MDD and control groups in the GSE92538 dataset was performed using the “limma” package (v 3.56.2) (28), aiming to identify differentially expressed genes (DEGs). Filtering was conducted with thresholds of *P*.adj < 0.05 and |log2Fold Change (FC)| > 0. Volcano plots and heat maps were generated using the “ggplot2” (v 3.5.2) (29) and “ComplexHeatmap” (v 2.16.0) (30) packages, respectively, to visualize the expression patterns of DEGs. The top 10 up-regulated and down-regulated DEGs, selected based on their Log2FC values, were highlighted in the volcano plot.

### 2.3 Weighted gene co-expression network analysis identified key module genes associated with MQC

To assess MQRG scores in MDD patients and controls within the training set, enrichment analysis was conducted using the “GSVA” software (v 1.48.2) (31). This analysis calculated the score for each sample, which served as phenotypic data for subsequent WGCNA. The significance of the variations in MQRG scores between the two groups was evaluated using the Wilcoxon rank-sum test (*P* < 0.05).

The “WGCNA” package (v 1.73) (32) was utilized to conduct weighted co-expression network analysis solely on the MDD samples from the training set (GSE92538). Hierarchical clustering was performed to identify outliers. To determine the gene-gene correlation threshold, a soft-thresholding power was selected from a range of 1 to 20, aiming for a scale-free fit index (R^2^) ≥ 0.85. The optimal soft-thresholding power was chosen to balance scale-free topology with biological relevance, resulting in a scale-free network. This network segregated genes into modules, with parameters defined as follows: minimum module size = 100 genes, module merging cut height = 0.2, and deep split sensitivity = 4. The correlation between module eigenvectors and ssGSEA scores was calculated to establish associations between scores and modules. Modules exhibiting the highest positive and negative correlations were selected (|correlation (cor)| > 0.3 and *P* < 0.05), and genes present in both modules were identified as key module genes, or MQRGs.

### 2.4 Acquisition of candidate genes and functional analysis

The “ggvenn” package (v 0.1.10) (33) was utilized to identify potential overlapping genes between DEGs and MQRGs_module. To facilitate sample-level comparisons with the microarray datasets, raw counts from the snRNA-seq dataset (GSE144136) were converted into pseudo-bulk expression profiles by aggregating counts at the sample level. This method aimed to evaluate whether the candidate biomarkers exhibited directionally consistent expression trends across platforms, while acknowledging that the pseudo-bulk transformation cannot entirely eliminate technical differences between microarray and snRNA-seq data. Differential expression analysis of candidate biomarkers between MDD and control groups within this pseudo-bulk matrix was conducted using the Wilcoxon rank-sum test. To minimize potential confounding effects, rigorous covariate adjustments were implemented for the differential expression analysis in the training cohort (GSE92538). Clinical metadata, including age, sex, post-mortem interval (PMI), and cerebellar pH, were retrieved from the GEO database, with samples lacking this metadata being excluded. A linear model was constructed using the model.matrix function, incorporating diagnosis status (MDD vs. Control) as the primary variable and age, sex, PMI, and pH as covariates to ensure unbiased estimation of gene expression changes. Gene Ontology (GO) and Kyoto Encyclopedia of Genes and Genomes (KEGG) enrichment analyses (*P* < 0.05) were performed using the “clusterProfiler” tool (v 4.10.1) (34) to explore biological functions and pathways. The top five significant terms in each GO category (cellular component, molecular function, biological process) and the top 15 significant terms in KEGG were visualized.

### 2.5 Construction and evaluation of 10 machine learning algorithm models

Ten machine learning algorithms were incorporated to ensure reliable models and analytical outcomes, including Logistic Regression (LR), Random Forest (RF), Decision Tree (DT), Bagged Tree (BT), Linear Discriminant Analysis (LDA), Support Vector Machine (SVM), Neural Network (NNET), Stochastic Gradient Boosting Tree (SGBT), Naive Bayes (NB), and K-Nearest Neighbor (KNN). To ensure reproducibility, a fixed random seed (set.seed(12345)) was used throughout the analysis. The “caret” package (v 7.0.1) (35) performed recursive feature elimination (RFE) to identify important features within GSE92538. During this process, 10-fold cross-validation was employed to assess model performance, enabling the determination of the optimal feature subset and variable importance for each model. Based on important variables selected by RFE combined with 10-fold cross-validation, disease prediction models were constructed using the mentioned algorithms. After model construction, results from each fold were merged. Hyperparameter tuning was not conducted; all algorithms utilized default parameters (e.g., RF: ntree = 500; KNN: k = 5; NNET: size = 5, decay = 0.01, maxit = 1000; SGBT: n.trees = 500, interaction.depth = 3, shrinkage = 0.01, bag.fraction = 0.7) to facilitate fair comparisons. The “pROC” package (v 1.18.5) (36) was employed to plot receiver operating characteristic (ROC) curves, and evaluation metrics, such as the area under the curve (AUC), were calculated (AUC > 0.7 indicated good predictive accuracy). The average results from each fold for each algorithm were computed, with the model exhibiting the highest AUC and strong performance across other metrics selected as the optimal model. Subsequently, the “fastshap” package (v 0.1.1) (37) was used to conduct SHAP evaluation on the optimal model. The contribution of each gene was quantified through SHAP values, with the top 10 genes showing the highest contributions identified as characteristic genes for subsequent analysis. Additionally, the dependency relationship between feature genes and SHAP values, as well as the identification of high-risk samples based on SHAP values of feature genes, were explored.

### 2.6 Identification of candidate MQC-associated biomarkers and exploratory functional enrichment analysis

The Wilcoxon test was conducted to analyze expression differences of characteristic genes between the MDD group and the control group across two datasets (GSE92538 and GSE54570). Genes exhibiting significant inter-group differences in both the training and validation sets were selected. Candidate biomarkers were identified as genes with consistent expression trends (*P* < 0.05). Additionally, ROC analysis on the candidate biomarkers was conducted using the “pROC” package (version 1.18.5) to calculate the AUC value. Genes with an AUC greater than 0.7 in both the training and validation sets were classified as candidate biomarkers (AUC > 0.7 indicates good predictive accuracy).

GSEA was performed to understand the functions of candidate biomarkers involved in the development of MDD. Spearman correlation coefficients were calculated using the cor function for each candidate biomarker in relation to all genes across all samples from the GSE92538 dataset. The genes were then ranked from highest to lowest based on correlation coefficients, producing an ordered list of genes associated with each candidate biomarker. Following this, GSEA analysis was executed using the “clusterProfiler” package (v 4.10.1). The Molecular Signatures Database (MSigDB) (https://www.gsea-msigdb.org/gsea/msigdb) was utilized as a resource, with the “msigdbr” tool (v 10.2) (38) employed to identify the gene set belonging to category C2 and subcategory KEGG as the reference gene set (*P*.adj < 0.05). The top five signaling pathways based on candidate biomarkers’ enrichment results were visualized in descending order of *P*.adj value. It is important to note that GSEA is a computational method that infers pathway enrichment based on gene expression rank correlations; it does not directly measure biochemical activity, mitochondrial respiratory function, oxygen consumption rate (OCR), or ATP generation. Therefore, all GSEA results should be interpreted as statistical associations that generate hypotheses for future functional validation.

### 2.7 GeneMANIA and subcellular localization analysis

To identify additional genes related to the functions of candidate biomarkers, the GeneMANIA database (http://genemania.org) was employed to predict genes associated with the functions of candidate biomarkers.

### 2.8 Preliminary mRNA-level verification of candidate biomarkers in the CRS rat model

#### 2.8.1 Animals

Adult male Sprague-Dawley rats (6–8 weeks old), obtained from Vital River Laboratory Animal Technology Co., Ltd. (Beijing, China), were maintained at room temperature (22 ± 2 °C) under a 12-hour light-dark cycle with unrestricted access to food and water. All experiments conducted on rats adhered to ethical guidelines, and the protocol was approved by the ethics committee of Beijing MedCona Biotechnology Co., Ltd (approval no. MDKN-2025-048). Rats were allocated to experimental groups using computer-generated random number sequences prior to modeling. All behavioral recordings, brain tissue dissection, and RNA isolation were performed by independent researchers who were blinded to the animal group assignments. No predefined exclusion criteria were applied, and no rats were excluded throughout the experiment due to illness or abnormal behaviors.

#### 2.8.2 Chronic restraint stress model

Rats were randomly assigned to two experimental groups: a control group (n = 8) that received no stress intervention and a chronic restraint stress (CRS) group (n = 8). Following one week of acclimation, rats in the CRS group were placed in custom-made restraint tubes for 6 hours daily at random time points. The CRS model was established after three consecutive weeks of stress exposure, with behavioral tests conducted post-modeling.

#### 2.8.3 Behavioral tests

Depressive-like behaviors were assessed using a battery of tests, including the Sucrose Preference Test (SPT), Open Field Test (OFT), and Forced Swim Test (FST), each preceded by a 30-minute acclimation period.

SPT: The SPT assessed anhedonia. After 24 hours of food and water deprivation, individually housed rats were given simultaneous access to a sucrose solution and plain water for 4 hours. To prevent place preference, the positions of the bottles were switched after 2 hours. Sucrose preference was calculated as (sucrose intake/(sucrose intake + water intake)) × 100%, with a reduction indicating anhedonia.

OFT: The OFT was performed in an open square arena (100 × 100 × 50 cm^3^), cleaned with 70% ethanol between sessions. Each rat was allowed 5 minutes for free exploration from the center point. Measurements included latency to enter the central zone, number of crossings, and frequency of rearing. Elevated crossings, rearings, or immobility in corners could indicate anxiety or depressive-like behavior.

FST: The FST was conducted in a transparent glass cylinder (25 cm diameter, 50 cm height) filled with water at 25 ± 1 °C to a depth of 35 cm. During the 5-minute test, immobility time was recorded, with increased immobility duration interpreted as behavioral despair.

#### 2.8.4 Real-time quantitative PCR

Six rats from each group were randomly selected for PFC collection and subsequent RT-qPCR detection. The remaining two rats per group were reserved for Nissl staining to observe CRS-induced neuronal damage; however, these histological data were not analyzed or reported in this manuscript, resulting in the exclusion of their tissues from the qPCR sample cohort. Following anesthesia, the rats were decapitated, and the entire brain was swiftly excised and placed on ice. The PFC was promptly dissected on a chilled surface based on anatomical landmarks and stored at −80 °C. Subsequently, frozen tissue samples were thawed on ice, and total RNA was isolated using TRIzol reagent (Invitrogen, USA). The RNA was then transcribed into cDNA using M-MLV reverse transcriptase (TaKaRa, China) following the manufacturer’s guidelines. Quantitative PCR was conducted with TB Green Premix Ex Taq II (TaKaRa, China) on a Roche LightCycler^®^ 480 II Real-Time PCR System (Roche, Switzerland), utilizing the primer sequences specified in **Table 1**. This experiment was designed as preliminary verification of expression levels rather than functional validation of MQC regulation.

**Table 1:** Primer sequences for qPCR.

Gene	Sequence (5’ to 3’)	Product length
GAPDH	F: TGCTGAGTATGTCGTGGAG	288 bp
	R: GTCTTCTGAGTGGCAGTGAT	
*DCHS1*	F: ACTACCGTGTTGCTGTGTCTGAA	125 bp
	R: CCGATTGCCACTGATAATGCTGTA	
*HS3ST2*	F: GCTGATAGTGGTGGTGCGGAAC	113 bp
	R: TGCGGTTGCGGAAGGACAAG	

### 2.9 snRNA-seq analysis

Initial data filtering of the GSE144136 dataset was performed using the “Seurat” package (v 5.3.0) (39) to focus the cellular-level gene expression analysis (MDD vs. control) on core cells and genes. The filtering criteria excluded genes detected in fewer than five cells, cells with fewer than 300 genes, cells expressing more than 6000 genes (indicating abnormally high expression), and cells with total mRNA counts exceeding 20, 000. As the original submitters of the GSE144136 dataset had removed all mitochondrial transcripts during upstream raw data processing, additional filtering based on the percentage of mitochondrial genes was not incorporated in our analysis pipeline. The DoubletFinder algorithm was employed to detect artificial doublet nuclei. Preliminary evaluations indicated that the doublet proportion within this dataset was extremely low, and removing doublets would not significantly alter cell clustering patterns, biomarker expression, or pathway activity; thus, all filtered nuclei were retained for subsequent unified analysis. The dataset encompassed 6 independent samples from the MDD group and 6 from the control group. This study reported only the global total number of nuclei before and after quality control rather than individual nucleus counts for each sample, as our primary research objectives centered on cross-group comparisons of cell-type proportions and gene expression rather than single-sample quantitative statistics. The Seurat function FindVariableFeatures was utilized to screen for the top 2, 000 highly variable genes (HVGs), highlighting the 10 most variable genes. Subsequently, the RunPCA function conducted a principal component analysis (PCA) on these HVGs. Key principal components (PCs) for further analysis were identified by integrating results from a Jackstraw permutation test (*P* < 0.05) and the plateau point in the scree plot. Unsupervised clustering was performed on the PCA results using the FindNeighbors and FindClusters functions (resolution = 0.1) to define distinct cell clusters. Cell type annotation relied on published marker genes (40) and data from the CellMarker 2.0 website (http://bio-bigdata.hrbmu.edu.cn/CellMarker/), complemented by an analysis of their expression across the identified clusters.

### 2.10 The activity level of MQRGs in single cells

To evaluate the activity level of the MQRGs gene set in single cells, the AUCell_buildRankings function was used to create a gene ranking matrix for each cell based on MQRGs expression levels. Following this, the AUCell_calcAUC function was applied to compute the AUC values of the MQRGs gene set in each cell, providing a computationally inferred enrichment score that reflects the enrichment level of gene set members within the top 10% of each cell’s gene expression rankings. The resulting AUC score matrix was subsequently integrated back into the Seurat object using the AddMetaData function.

### 2.11 Enrichment analysis and cell communication

Our investigation into the biological pathways, functions, and cellular communication networks underlying the progression of MDD employed two distinct computational approaches on the GSE144136 dataset. First, functional enrichment across various cell types was assessed using the “ReactomeGSEA” package (v 1.16.1) (41) (*P* < 0.05). Second, the “CellChat” package (v 1.6.1) (42) was utilized to analyze ligand-receptor pairs and intercellular interactions. We employed the curated CellChatDB.human database, focusing specifically on secreted signaling pathways. To minimize noise from rare populations, interactions involving cell groups with fewer than three cells were filtered out (min.cells = 3). Communication probabilities were inferred through permutation tests, and significant interactions were identified using thresholds of *P* < 0.05 and Log2mean(Molecule1, Molecule2) ≥ 0.1.

### 2.12 Pseudo-time and transcription factor analysis

Dimensionality reduction and clustering analysis of key cells were performed utilizing the methodology outlined in section 2.11. PCs (*P* < 0.05) were filtered and obtained through the “Seurat” package (v 5.3.0). Following sorting, these PCs were selected as valid components for subsequent analyses. Cell subpopulations of key cells were annotated based on marker genes for each cell cluster derived from the FindAllMarkers function. Pseudotime trajectory analysis was conducted on key cells after dimensionality reduction and subclustering, with the “Monocle3” package (v 2.30.1) (43) used to plot the dynamic trends of candidate biomarkers’ contributions along the pseudotime trajectory, reflecting transcriptional state dynamics. It is important to note that in the context of adult human post-mortem brain tissue, pseudotime trajectories inferred from snRNA-seq data typically represent dynamic changes in transcriptional states or cellular activity rather than true lineage differentiation or developmental progression.

### 2.13 Statistical analysis

Data analysis was conducted using R software (v 4.2.2) and SPSS 20.0 software. The Wilcoxon test evaluated differences between groups. The Shapiro-Wilk test was employed to assess the normality of the experimental data, followed by one-way analysis of variance (one-way ANOVA) for statistical analysis. A *P*-value of less than 0.05 was considered statistically significant.

## 3 Results

**Figure 1:**
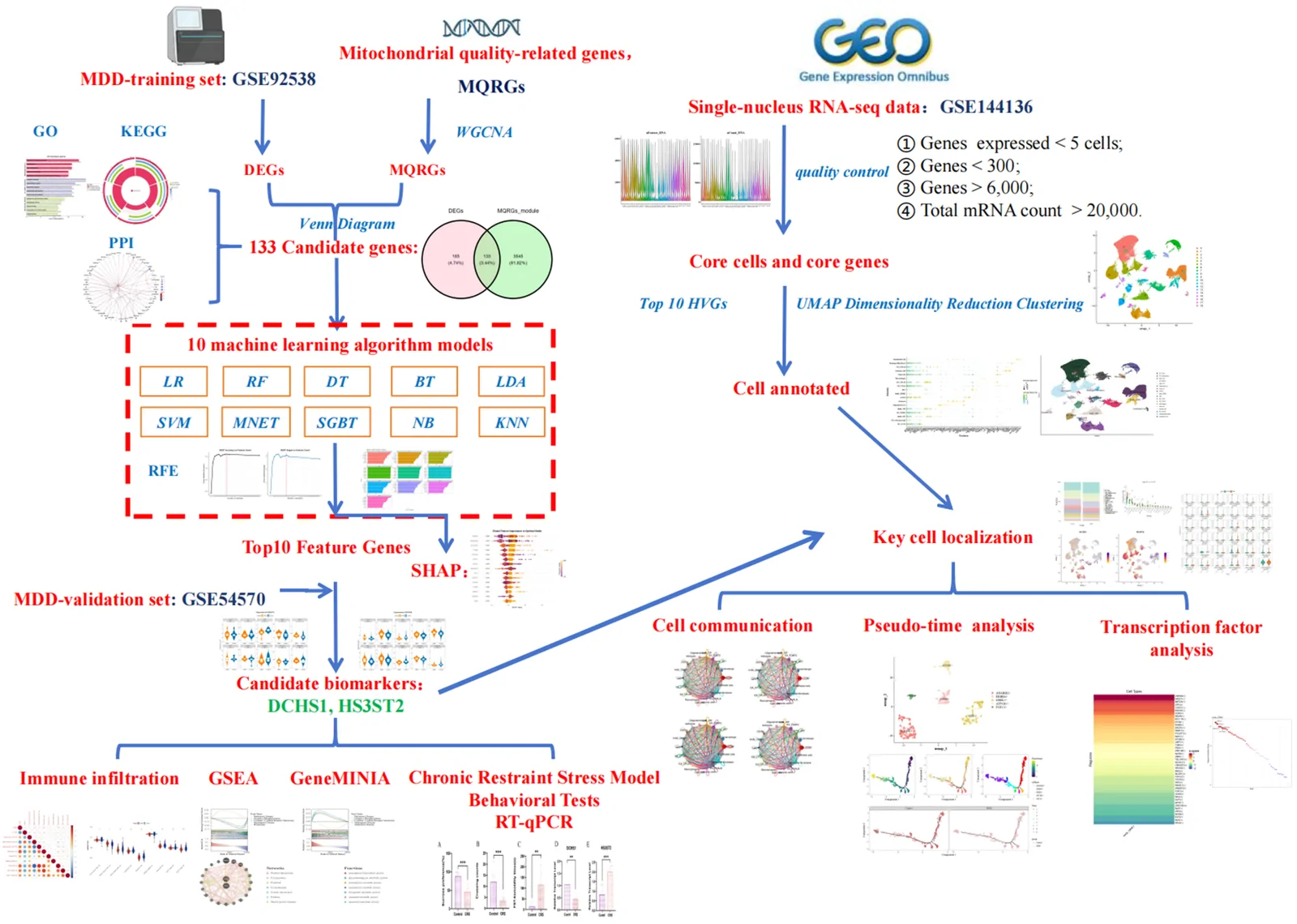
Schematic workflow.

The overall study and experimental workflow are illustrated in **Figure 1**.

### 3.1 Screening and functional analysis of candidate genes regulating mitochondrial quality in depression

**Figure 2:**
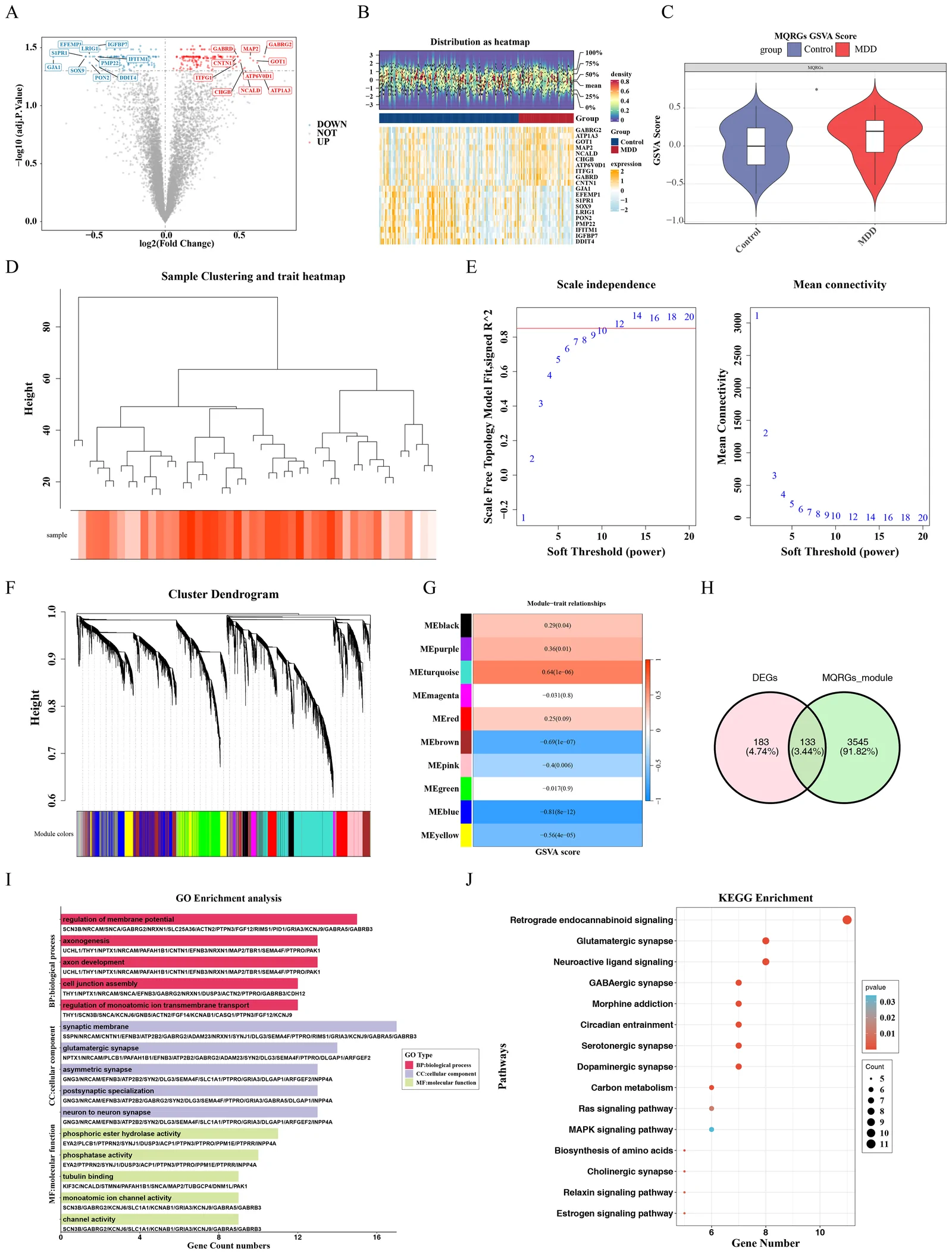
Integrative analysis identifies candidate genes associated with mitochondrial quality control (MQC) in MDD. **(A)** Volcano plot of differentially expressed genes (DEGs) in the PFC of MDD patients versus controls, with red dots indicating up-regulated genes and blue dots indicating down-regulated genes. **(B)** Heatmap displaying the expression patterns of the top 50 DEGs. **(C)** Boxplot comparing MQRG scores between MDD and control groups. **(D)** Hierarchical clustering results. **(E)** Selection of the soft-thresholding power (β = 12) for WGCNA. **(F)** Dendrogram of co-expression modules. **(G)** Correlation heatmap between module eigengenes and MQRG scores. **(H)** Venn diagram illustrating the intersection of 316 DEGs (from A) and 3, 678 MQC-related module genes (from G), resulting in 133 candidate genes for downstream analysis. **(I, J)** Functional enrichment analysis of the 133 candidate genes.

A total of 316 DEGs were identified between the MDD and control groups in the GSE92538 dataset. Among these DEGs, 179 genes exhibited significant up-regulation in the MDD group compared to the control group, while 137 genes showed significant down-regulation (*P*.adj < 0.05, |log2FC| > 0) (**Figures 2, B**, **Supplementary Table 1**).

A significant difference in MQRG scores was observed between the MDD and control groups (*P* < 0.05), indicating the high potential of MQRGs for exploration in MDD (**Figure 2C**). Hierarchical clustering revealed no outliers. Thus no samples were excluded (**Figure 2D**). The optimal soft threshold of 12 (R^2^ = 0.877) was determined through scale independence and mean connectivity analyses (**Figure 2E**). WGCNA identified 10 co-expression modules (**Figure 2F**). Correlation analysis (|cor| > 0.3, *P* < 0.05) between modules and ssGSEA scores demonstrated that the “turquoise” module had the strongest positive correlation (cor = 0.64, *P* < 0.05), while the “blue” module exhibited the strongest negative correlation (cor = -0.81, *P* < 0.05) (**Figure 2G**). These two modules, collectively comprising 3, 678 genes, were defined as MQRGs_module (**Supplementary Table 2**).

The DEGs were intersected with the MQRGs_module, which revealed 133 candidate genes (**Figure 2H**, **Supplementary Table 3**). Functional enrichment analysis characterized the biological functions and signaling pathways associated with these genes. This analysis revealed significant enrichment in 545 GO entries, encompassing 382 biological processes (BPs), 82 cellular components (CCs), and 81 molecular functions (MFs) (*P* < 0.05) (**Supplementary Table 4**). Notable entries included axon development, synaptic membrane, and tubulin binding (**Figure 2I**). Additionally, the candidate genes were significantly enriched in 40 KEGG signaling pathways, including retrograde endocannabinoid signaling, glutamatergic synapse, neuroactive ligand signaling, GABAergic synapse, serotonergic synapse, dopaminergic synapse, carbon metabolism, Ras signaling pathway, and MAPK signaling pathway (*P* < 0.05) (**Figure 2J**, **Supplementary Table 5**). These pathways are closely linked to the pathogenesis of MDD, involving metabolic pathways and signaling cascades related to energy metabolism.

### 3.2 Construction and evaluation of 10 machine learning algorithm models

**Figure 3:**
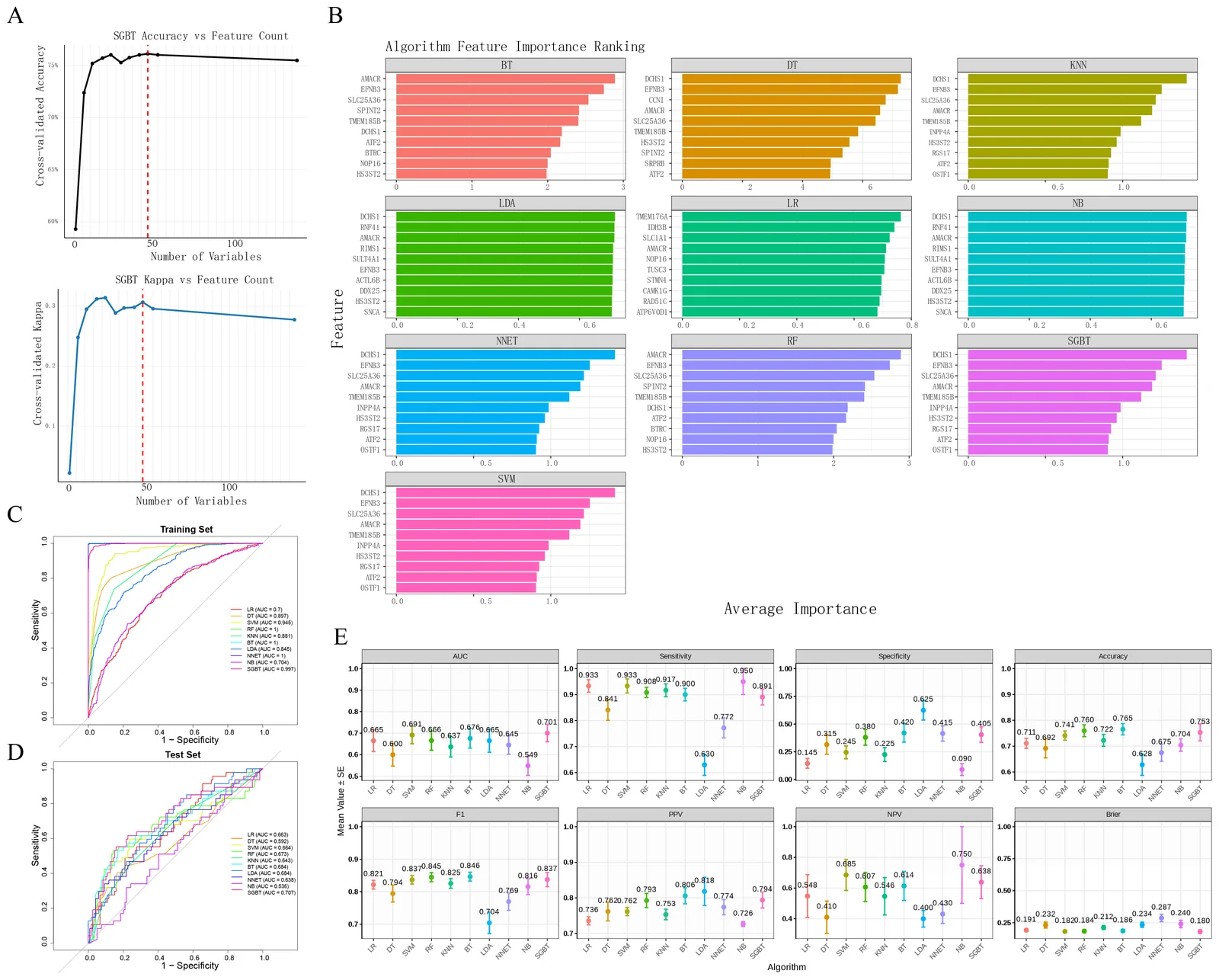
Construction and evaluation of ten machine learning models for MDD classification. **(A)** Line plot of feature selection using the RFE algorithm: the variable screening of the SGBT algorithm. The horizontal axis represents the number of variables, while the vertical axis displays accuracy and Kappa values calculated via 10-fold cross-validation. The red dotted line indicates the optimal number of variables. **(B)** Variable importance ranking across different machine learning algorithms, with DCHS1 (red arrow) consistently identified as a top contributor. **(C, D)** Receiver Operating Characteristic (ROC) curves with 10-fold cross-validation for all ten algorithms in the training set. **(E)** Radar chart comparing performance metrics (Accuracy, Precision, Recall, F1-score) of the top three models (SGBT, LDA, BT), with SGBT selected as the optimal model.

Using the RFE method (**Supplementary Figures 1A–I**), optimal feature subsets for each machine learning model were identified. Notably, in the variable screening of the SGBT algorithm (**Figure 3A**), the model achieved the highest accuracy and Kappa value when incorporating 45 variables. Among the ten algorithms evaluated, *DCHS1* consistently ranked as the most important gene (**Figure 3B**). ROC curve analyses indicated that, ranked by AUC values, the top three algorithms were SGBT (0.707), LDA (0.684), and Bagging Trees (BT) (0.684), with an AUC > 0.7 indicating good predictive accuracy (**Figures 3C, D**). To rigorously evaluate the statistical uncertainty of these estimates, we calculated the 95% confidence intervals (CIs) for the AUC of each algorithm across the 10 folds (**Supplementary Table 6**). Among them, the SGBT algorithm achieved the highest AUC in the test set and performed exceptionally well in accuracy, precision, recall, and F1 score, leading to its selection as the optimal model (**Figure 3E**, **Table 2**).

**Table 2:** Training and test sets log loss.

Model	Log loss train	Log loss test
SVM	0.370684	0.5495
LR	0.543878	0.562303
SGBT	0.168062	0.570366
RF	0.137395	0.571854
BT	0.135963	0.577614
LDA	0.480509	0.684552
NNET	0.019792	1.717832
NB	1.510064	2.163207
DT	0.336512	2.247625
KNN	0.367768	3.097969

### 3.3 Identification and exploratory functional analysis of candidate MQC-associated biomarkers

**Figure 4:**
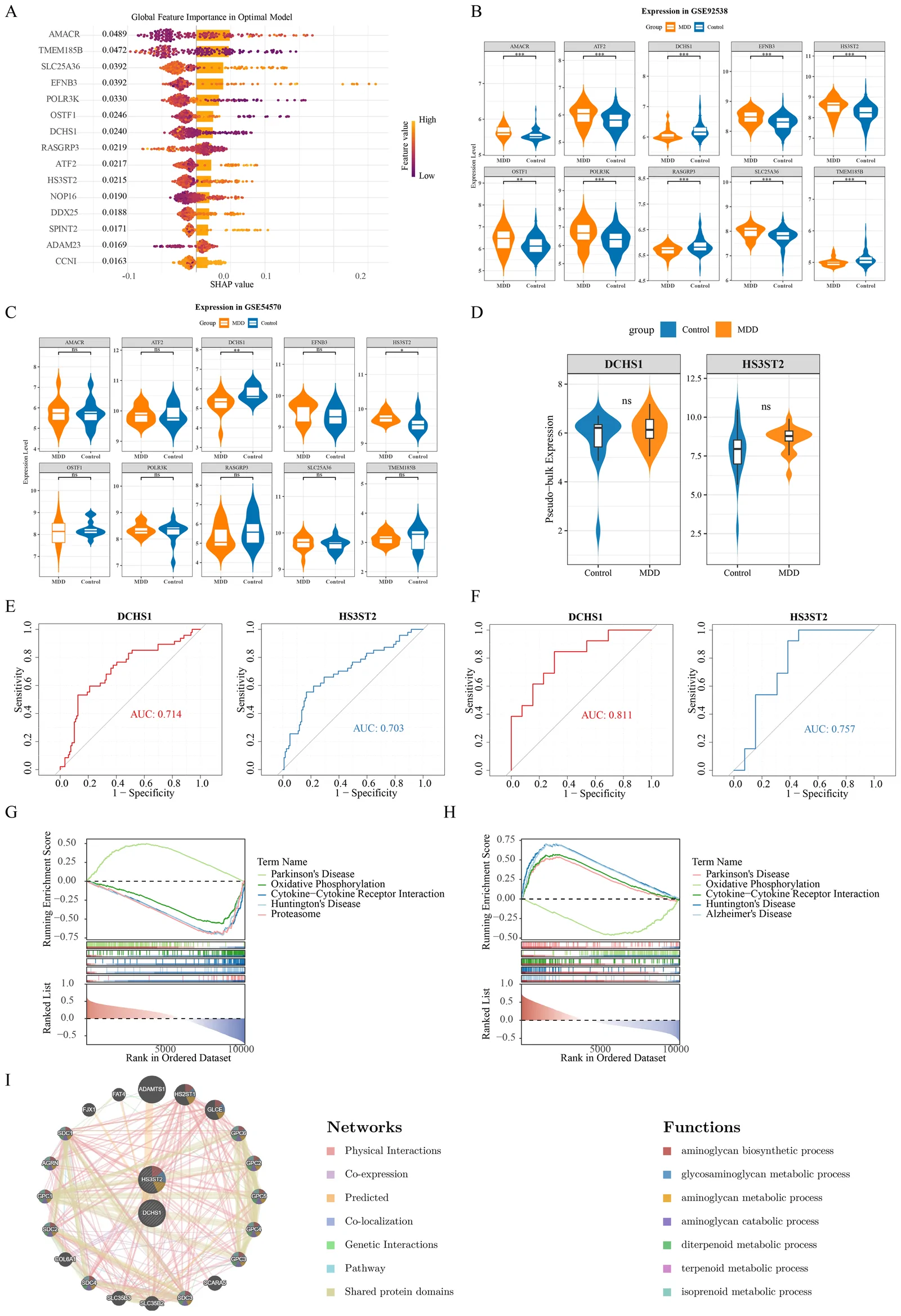
**(A)** SHAP (Shapley Additive exPlanations) summary plot illustrating the global importance of the top ten genes for disease prediction in the SGBT model. **(B, C)** Expression levels of disease targets in the training and validation sets (**B**: Training set; **C**: Validation set). **(D)** Comparative analysis of DCHS1 and HS3ST2 expression in the microarray training set and pseudo-bulk validation set. **(E, F)** ROC curves demonstrating the high discriminative ability of DCHS1 and HS3ST2 in both the training and validation cohorts (AUC > 0.7) (**E**: Training set; **F**: Validation set). **(G, H)** Gene Set Enrichment Analysis (GSEA) showing that DCHS1 and HS3ST2 are enriched in the oxidative phosphorylation (OXPHOS) and cytokine-cytokine receptor interaction pathways. **(I)** GeneMANIA network predicting functional interactions of DCHS1 and HS3ST2 with other genes involved in aminoglycan biosynthesis.

SHAP model evaluation identified the top ten genes contributing to the optimal SGBT model, ranked as follows: *AMACR*, *TMEM185B*, *SLC25A36*, *EFNB3*, *POLR3K*, *OSTF1*, *DCHS1*, *RASGRP3*, *ATF2*, and *HS3ST2*. These genes were designated as characteristic genes for further analyses. Among them, high expression of *AMACR*, *SLC25A36*, *EFNB3*, *ATF2*, *HS3ST2*, *NOP16*, *DDX25*, SP*I*NT2, *ADAM23*, and *CCNI* significantly increased SHAP values, suggesting that their elevated expression correlates positively with disease occurrence. In contrast, low expression of *TMEM185B*, *POLR3K*, *OSTF1*, *DCHS1*, and *RASGRP3* was associated with a decreased risk of disease, exhibiting a negative correlation with disease occurrence (**Figure 4A**).

Further expression analyses in the GSE92538 and GSE54570 datasets indicated that *DCHS1* and *HS3ST2* exhibited intergroup differences, demonstrating consistent expression trends across both datasets, leading to their identification as candidate biomarkers (*P* < 0.05) (**Figures 4B, C**). Although direct comparisons within the snRNA-seq-derived pseudo-bulk dataset did not reach statistical significance (*P* > 0.05), likely due to limited sample size and statistical power, *DCHS1* and *HS3ST2* displayed directionally consistent expression trends compared to those in the microarray datasets (GSE92538 and GSE54570). These findings provide supportive, albeit not definitive, cross-platform evidence for their potential relevance as MDD-associated candidate biomarkers (**Figure 4D**). To address potential confounders, we reanalyzed GSE92538 by incorporating age, sex, PMI, and pH as covariates in the limma model. Notably, the core genes *DCHS1* and *HS3ST2* retained their expression trends and statistical significance (*DCHS1*: *P*.adj < 0.05, |log2FC| = -0.104; *HS3ST2*: *P*.adj < 0.05, |log2FC| = 0.160) (**Supplementary Table 7**), confirming their robustness against clinical bias. Additionally, *DCHS1* and *HS3ST2* exhibited AUC values above 0.7 in both training and validation datasets, highlighting their proficiency in differentiating MDD samples. Consequently, they were designated as candidate biomarkers (**Figures 4E, F**).

The GSEA enrichment analysis revealed that *DCHS1* and *HS3ST2* were enriched in 63 (**Figure 4G**, **Supplementary Table 8**) and 58 (**Figure 4H**, **Supplementary Table 9**) pathways, respectively, with notable associations including OXPHOS and cytokine-cytokine receptor interaction (*P*.adj < 0.05). These computational predictions suggest potential functional linkages but do not provide biochemical evidence of altered pathway activity. **Figure 4I** illustrates that the GeneMANIA analysis demonstrated high connectivity of the candidate biomarkers with ADAMTS1, HS2ST1, GLCE, and other genes, indicating their significant roles in MDD-related functional modules. Functional analysis of this network highlighted the involvement of these genes in various biological processes, including the aminoglycan biosynthetic process.

### 3.4 Preliminary expression verification of candidate biomarkers in the CRS rat model

**Figure 5:**
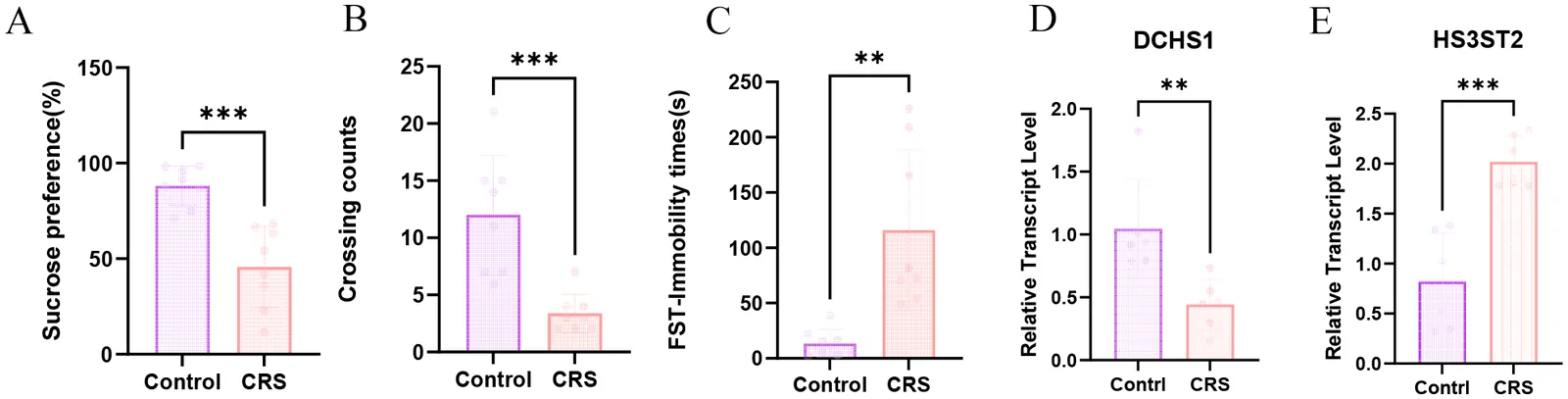
Preliminary mRNA-level verification of candidate biomarkers in the CRS rat model. **(A)** Sucrose Preference Test (SPT) showing decreased anhedonia in CRS-treated rats compared to controls (n = 8). **(B)** Open Field Test (OFT) showing reduced central zone crossings in CRS-treated rats (n = 8). **(C)** Forced Swim Test (FST) showing increased immobility time in CRS-treated rats (n = 8). **(D)** The mRNA expression of *DCHS1* in the PFC of CRS-treated rats was significantly down-regulated compared to the control group (n = 6). **(E)** The mRNA expression of *HS3ST2* in the PFC of CRS-treated rats was significantly up-regulated compared to the control group (n = 6). **P < 0.01. ***P < 0.001.

Given the significance of *DCHS1* and *HS3ST2* identified in this study, we selected these two candidate biomarkers for further experimental validation. Initially, we established a CRS rat model of depression and conducted behavioral evaluations. Compared to the control group, the CRS group exhibited a decreased sucrose preference rate, increased immobility time in the FST, and a reduced number of central zone crossings in the OFT (**Figures 5A–C**). mRNA analysis via qPCR in the rat PFC revealed that *DCHS1* expression was significantly down-regulated compared to the control group, while *HS3ST2* mRNA levels were significantly elevated (*P* < 0.05) (**Figures 5D, E**).

### 3.5 snRNA-seq-based MQRG activity analysis and identification of key cell types

**Figure 6:**
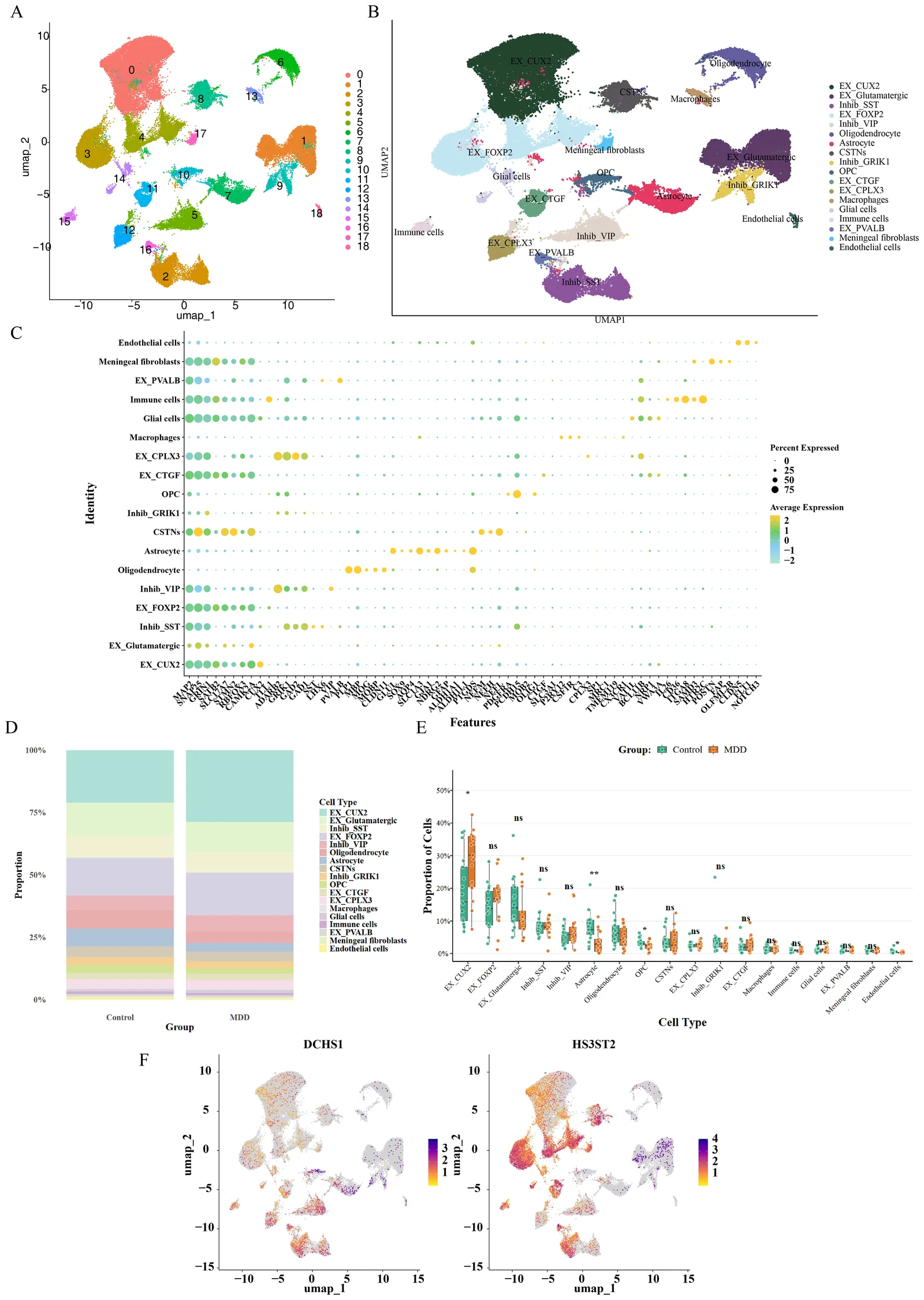
snRNA-seq-based cellular landscape of the PFC. **(A)** UMAP plot illustrating unsupervised clustering of 78, 295 single nuclei from the PFC, colored by identified cell clusters. **(B)** UMAP plot showing cell type annotation, including excitatory neurons (EX), inhibitory neurons (Inhib), and glial cells. **(C)** Dot plot of annotated cell clusters; the x-axis represents marker genes, the y-axis shows different cell clusters; color intensity indicates expression level, with yellow representing higher expression, and dot size reflects the proportion of cells expressing the gene. **(D)** Cell type abundance plot; the x-axis represents relative abundance of each cell type in the control and disease groups. **(E)** Boxplot showing differences in cell type proportions between groups. **(F)** UMAP plot visualizing the expression of candidate biomarkers. .

Single-nucleus RNA sequencing (snRNA-seq) data analysis revealed a total of 78, 343 cells and 28, 211 genes detected before quality control (**Supplementary Figure 2A**), while 78, 295 cells and 28, 211 genes were retained post-quality control (**Supplementary Figure 2B**). The top ten genes with the highest expression variation included RELN, CXCL14, HTR2C, among others (**Supplementary Figure 2C**). The first 20 statistically significant PCs (*P* < 0.05) were selected for subsequent analysis (**Supplementary Figures 2D, E**), and unsupervised clustering analysis identified 19 distinct cell clusters (**Figure 6A**). A total of 18 cell types were annotated, including endothelial cells, EX_PVALB, meningeal fibroblasts, glial cells, immune cells, macrophages, EX_CTGF, EX_CPLX3, oligodendrocyte precursor cells (OPCs), Inhib_GRIK1, corticothalamic neuron subsets (CSTNs), Inhib_VIP, astrocytes, oligodendrocytes, Inhib_SST, EX_Glutamatergic, EX_FOXP2, and EX_CUX2 (**Figures 6B, C**, **Table 3**). Both excitatory and inhibitory neurons exhibited subtype-specific gene expression patterns; for instance, Inhib_GRIK1 highly expressed ADARB2, GRIK1, GAD2, and GAD1, but lacked VIP and SST, identifying it as part of the inhibitory neuron population. Moreover, the number of cell types identified in this study was lower than that reported in previous research (26 cell types) (40), which is speculated to be due to inconsistent cell screening criteria employed in the analyses.

**Table 3:** Annotation of the cell types according to the marker genes.

No.	Cell type	Cluster	Marker gene
1	Endothelial cells	18	“CLDN5”, “FLT1”, “NOTCH3”
2	EX_PVALB	16	“PVALB”
3	Meningealfibroblasts	17	“POSTN”, “FAP”, “OLFML2B”
4	Glial cells	14	“VAT1L”, “BCL11B”, “NRP1”, ‘VWA3A’
5	Immunecells	15	“IL26”, “CD36”, “ITGA8”, “SAMD3”, “HTR2C”
6	Macrophages	13	“SP1”, “MRC1”, “TMEM119”, “CX3CR1”
7	EX_CTGF	11	“MAP2”, “SNAP25”, “GRIN1”, “SATB2”, “SLC17A7”, “STMN2”, “RBFOX3”, “CAMK2A”
8	EX_CPLX3	12	“P2RY12”, “CSF1R”, ‘C3’, “CPLX3”
9	OPC	10	“PDGFRA”, “PCDH15”, “MOB2”, “OLIG1”
10	Inhib_GRIK1	9	“ADARB2”, “GRIK1”, “GAD2”, “GAD1”
11	CSTNs	8	“NEFM”, “NEFH”, “NEFL”
12	Inhib_VIP	5	“ADARB2”, “GRIK1”, “GAD2”, “GAD1”, “VIP”
13	Astrocyte	7	“GLUL”, “SOX9”, “AQP4”, “SLC1A3”, “GJA1”, “NDRG2”, “GFAP”, “ALDH1A1”, “ALDH1L1”, “PTGDS”
14	Oligodendrocyte	6	“PLP1”, “MBP”, “MOG”, “MOBP”, “CLDN11”
15	Inhib_SST	2	“ADARB2”, “GRIK1”, “GAD2”, “GAD1”, “SST”
16	EX_Glutamatergic	1	“MAP2”, “SNAP25”, “GRIN1”, “SATB2”, “SLC17A7”, “STMN2”, “RBFOX3”, “CAMK2A”,
17	EX_FOXP2	3, 4	“MAP2”, “SNAP25”, “GRIN1”, “SATB2”, “SLC17A7”, “STMN2”, “RBFOX3”, “CAMK2A”, “FOXP2”
18	EX_CUX2	0	“MAP2”, “SNAP25”, “GRIN1”, “SATB2”, “SLC17A7”, “STMN2”, “RBFOX3”, “CAMK2A”, “CUX2”

To further identify key cell types within the single-cell data, we first analyzed the proportional distribution of annotated cell types between the MDD and control groups. The results indicated that the proportion of EX_CUX2 was highest in the MDD group, followed closely by EX_FOXP2 (**Figure 6D**). Subsequent intergroup differential analysis of cell abundance revealed significant differences in the abundance of EX_CUX2, astrocytes, OPCs, and endothelial cells between the two groups (*P* < 0.05), consistent with findings from previous studies (40) (**Figure 6E**).

The enrichment of MQRGs was further evaluated at the single-cell level using the AUCell algorithm. The distribution of MQRG enrichment scores across single cells indicated that cell types such as CSTNs, Inhib_SST, and EX_FOXP2 exhibited relatively high enrichment scores (**Supplementary Figure 2F**). Intergroup analysis of MQRG enrichment scores revealed substantial heterogeneity in the inferred MQRG enrichment across cell types, with significantly decreased enrichment scores observed in eight cell types from the MDD group, including EX_CUX2, Inhib_SST, EX_FOXP2, Inhib_VIP, CSTNs, OPCs, EX_CPLX3, and glial cells. This reduction suggests diminished activity of MQRGs in a variety of glial and neuronal cell types within the PFC of MDD patients (**Supplementary Figure 2G**).

Analysis of key MQRG expression within each cell cluster demonstrated significant differences in the expression of *DCHS1* and *HS3ST2* specifically in the inhibitory neuron Inhib_GRIK1 (*P* < 0.05) (**Figure 6F**, **Supplementary Figure 3**). Additionally, *HS3ST2* showed high expression in immune-related cells, such as macrophages, and was also detected in EX_CTGF, an excitatory neuron involved in the secretion of connective tissue growth factor and participation in neural development. This suggests that *HS3ST2* may play roles in immune responses and neural development. Based on these findings, Inhib_GRIK1 was identified as a key cell type for subsequent analysis.

### 3.6 Enrichment analysis and cell communication

To elucidate the relationship between the candidate biomarker-related cell type, Inhib_GRIK1, and MQC, we performed enrichment analysis and cell-cell communication analysis on key cell types in this study.

**Figure 7:**
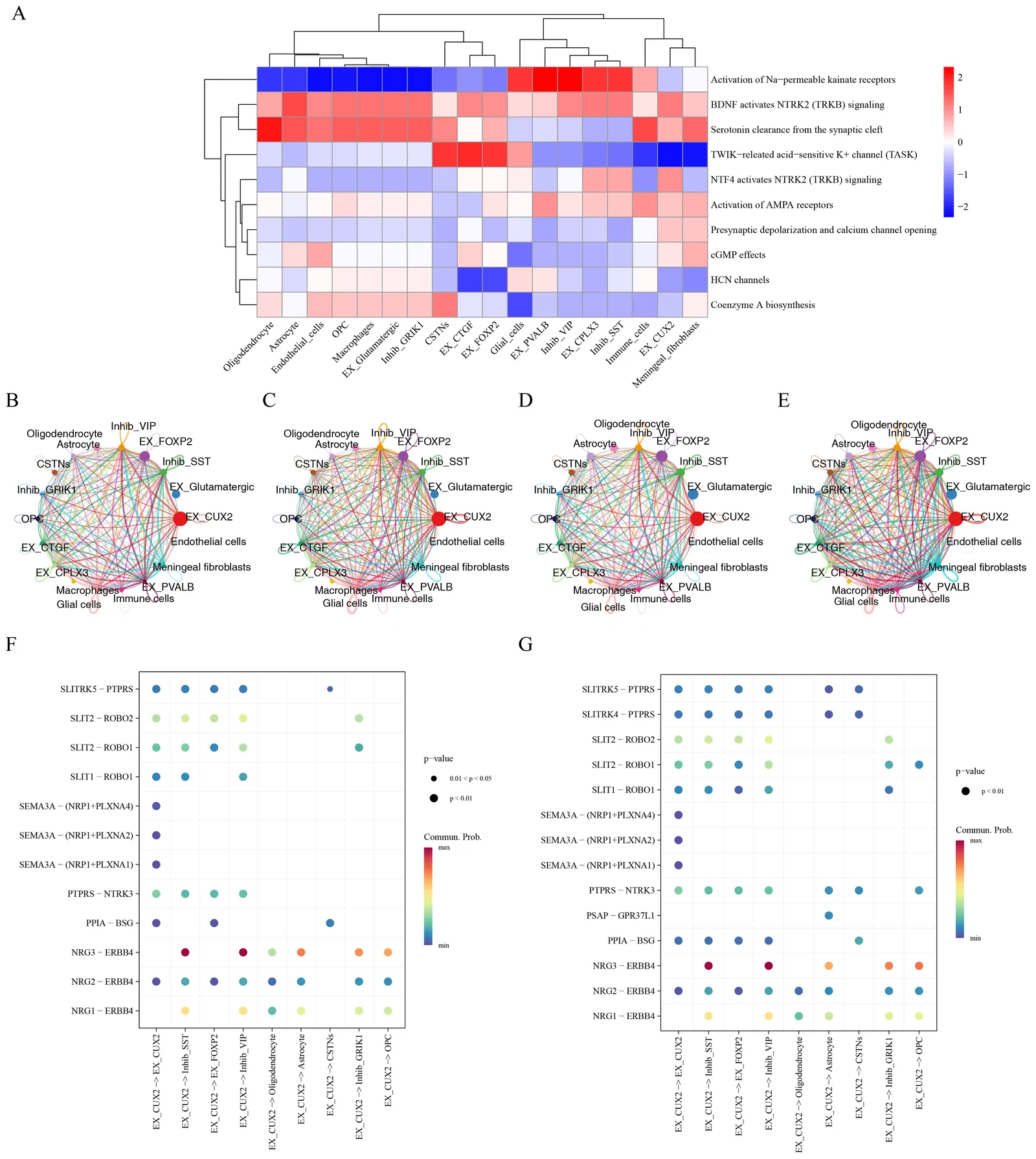
Functional enrichment and cell-cell communication analysis centered on Inhib_GRIK1 neurons. **(A)** Reactome pathway enrichment analysis across different cell types. **(B, C)** Circle plots illustrating outgoing signaling patterns of the Inhib_GRIK1 cluster in the MDD group. **(D, E)** Circle plots demonstrating incoming signaling patterns of the Inhib_GRIK1 cluster in the control group. **(F)** Bubble plot of significant ligand-receptor interactions in the MDD group. **(G)** Bubble plot of significant ligand-receptor interactions in the control group. The NRG3-ERBB4 pair exhibits the highest communication probability.

The enrichment analysis revealed significant pathway differences between the MDD and control groups, including the activation of AMPA receptors, serotonin clearance from the synaptic cleft, and activation of Na^+^-permeable kainate receptors (*P* < 0.05). Notably, astrocytes, macrophages, endothelial cells, and the key cell type Inhib_GRIK1 were significantly enriched in three core signaling pathways: “Activation of Na^+^-permeable kainate receptors, “ “BDNF activates NTRK2 (TRKB) signaling, “ and “Serotonin clearance from the synaptic cleft.” In contrast, excitatory neurons (EX_CUX2) were specifically and significantly enriched in the “BDNF activates NTRK2 (TRKB) signaling” and “TWIK-related acid-sensitive K^+^ channels (TASK)” pathways (**Figure 7A**).

Cell-cell communication analysis indicated that Inhib_GRIK1 had a close association with meningeal fibroblasts in both the MDD and control groups (*P* < 0.05, Log2mean (Molecule1, Molecule2) ≥ 0.1). Additionally, eight cell types demonstrated communication with Inhib_GRIK1: EX_CUX2, EX_FOXP2, and CSTNs exhibited unidirectional communication, while Inhib_SST, Inhib_VIP, OPCs, EX_CPLX3, and glial cells displayed bidirectional communication, suggesting potential synergistic effects among these cells (**Figures 7B–E**).

Furthermore, the CellChat package was employed to computationally infer ligand-receptor interactions between cells in the PFC tissues of MDD patients and normal controls. Results indicated that the NRG3−ERBB4 communication pathway exhibited the highest inferred communication probability in both groups. These probabilities represent transcriptome-based statistical estimates of ligand-receptor co-expression rather than direct measurements of pathway activity. Within this pathway, the highest communication probabilities were observed in EX_CUX2→Inhib_VIP and EX_CUX2→Inhib_SST interactions, while EX_CUX2→Astrocyte, EX_CUX2→Inhib_GRIK1, and EX_CUX2→OPC also demonstrated relatively high probabilities, suggesting that EX_CUX2 serves as a major signaling source in the cell communication network (**Figures 7F, G**).

### 3.7 Pseudo-time and transcription factor analysis

**Figure 8:**
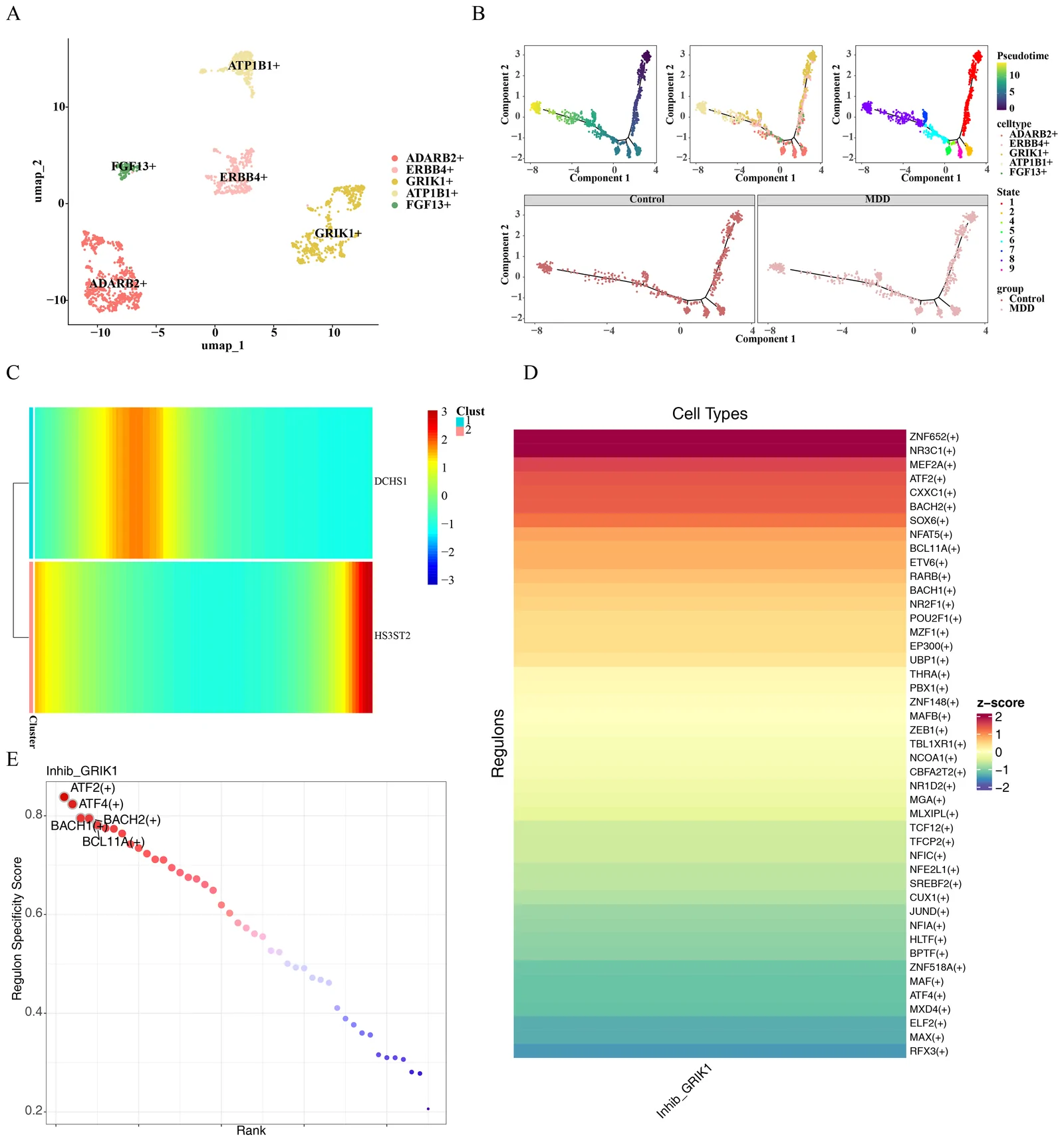
Pseudotime trajectory and transcriptional regulation analysis of Inhib_GRIK1 neurons. **(A)** UMAP plot of Inhib_GRIK1 cell subclusters, colored by subcluster identity. **(B)** Pseudotime trajectory analysis showing the inferred dynamic progression of Inhib_GRIK1 cells, ranging from early (light) to late (dark) states. **(C)** Heatmap illustrating the expression dynamics of *DCHS1* and *HS3ST2* along the pseudotime trajectory. *DCHS1* peaks in early/middle states, while *HS3ST2* peaks in early/late states. **(D)** Heatmap of transcription factor (TF) activity scores across Inhib_GRIK1 subclusters. **(E)** Ranking of transcription factors based on their regulatory specificity score within Inhib_GRIK1 cells.

For the dimensionality-reduced Inhib_GRIK1 cells, the top ten PCs were selected for subsequent analyses (*P* < 0.05) (**Supplementary Figures 4A–D**). These cells were subsequently divided into seven subclusters, annotated into five distinct cell types, all identified as ADARB2^+^, ERBB4^+^, GRIK1^+^, ATP1B1^+^, and FGF13^+^ (**Figure 8A**). Pseudotime ordering revealed nine distinct transcriptional states within the Inhib_GRIK1 population, with the majority of cells occupying the early and late states (1 and 8) (**Figure 8B**). The candidate biomarker *DCHS1* exhibited active expression (peaking) in the early to middle transcriptional states, while remaining inactive in other states. In contrast, *HS3ST2* was actively expressed in the early and late transcriptional states but inactive in other phases (**Figure 8C**).

Single-cell transcription factor analysis indicated that the transcription factors with the highest regulatory specificity scores (RSS) in Inhib_GRIK1 cells were ZNF652, NR3C1, MEF2A, ATF2, and CXXC1 (**Figure 8D**). According to the regional specificity score ranking, ATF2(+), ATF4(+), BACH1(+), BACH2(+), and BCL11A(+) demonstrated strong region-specific regulatory effects (**Figure 8E**).

## 4 Discussion

Imbalance in MQC is a critical factor in the pathogenesis of depression; however, the regulatory mechanisms and potential biomarkers remain unclear. This study employed transcriptome sequencing, snRNA-seq, and machine learning algorithms to investigate the correlations between MQC-related genes and activity alterations in depression. Cell subtypes exhibiting aberrant mitochondrial quality profiles in MDD were precisely identified. Notably, two genes with abnormal expression patterns in MDD were statistically linked to MQC processes. Differential expression of these genes was confirmed using qPCR in the PFC of rats with CRS-induced depression, supporting their potential utility as candidate diagnostic biomarkers and providing preliminary insights into the association between MDD and mitochondrial quality regulation. These previously unreported findings illustrate that machine learning algorithms significantly enhanced the stability and reliability of the gene screening pipeline, ensuring that the identified genes possess both statistical significance and potential biological relevance to depression pathogenesis. Collectively, these gene signatures offer novel, hypothesis-generating insights into the molecular correlates underlying depression.

*DCHS1* (Dachsous Cadherin-Related 1), a member of the protocadherin gene family, functions as both a cell adhesion and polarity establishment molecule, as well as an important regulator of neural progenitor cell differentiation and neuronal migration (44, 45). Variation or downregulation of *DCHS1* correlates with altered neural progenitor morphology, abnormal neuronal migration dynamics, and impaired autophagic processes (44–46). Notably, time-series analysis conducted in this study revealed that *DCHS1* was actively expressed in the early to middle transcriptional states of the key cell type Inhib_GRIK1. A recent study using cerebral organoids (hCOs) derived from patients carrying *DCHS1* mutations demonstrated that loss of *DCHS1* function results in increased spontaneous neuronal firing rates, decreased firing thresholds, and changes in neuronal morphological complexity and synaptic protein content (47). These observations suggest that alterations in *DCHS1*-associated neuronal excitability may represent a shared pathological correlate across neurodevelopmental defects, epilepsy, and mood disorders, although direct causal contributions to MDD remain to be validated.

*HS3ST2* (Heparan sulfate 3-O-sulfotransferase 2) is an enzyme that modifies heparan sulfate (HS) by catalyzing the production of 3-O-sulfated domains. This modification is clinically significant, as it influences HS interactions with various proteins, including growth factors and cytokines, which are critical for regulating cell signaling and inflammation (48). Previous studies have demonstrated that functional enhancement of *HS3ST2* can induce autonomous aggregation of tau protein in cells expressing the tauP301S mutation, as well as in those with wild-type tau protein (48, 49). In the brain tissues of Alzheimer’s disease (AD) patients, tau protein aggregation and succinylation levels increase, while succinylation levels of various mitochondrial proteins decrease (50). Notably, these tau-related findings were derived from AD models; the present study did not assess tau phosphorylation, aggregation status, or succinylation levels in MDD models or patient samples. In this study, the activities of MQC-related genes, such as those in OPCs, were found to decrease, closely related to abnormal protein aggregation in oligodendrocytes observed in neurodegenerative diseases (51). High expression of the *HS3ST2* gene was identified in the PFC of MDD patients and specifically in Inhib_GRIK1 and macrophages, with significant differences noted. Although direct evidence linking *HS3ST2* to depression remains lacking, existing literature—primarily from AD research—supports the hypothesis that *HS3ST2*-mediated protein aggregation may correlate with MDD pathogenesis by modulating neuroinflammation and mitochondrial functional homeostasis (50, 52, 53). However, this hypothesis requires validation through direct measurement of tau phosphorylation and aggregation levels in MDD models or patient samples.

Collectively, the multi-omics and machine learning analyses conducted in this study suggest that *DCHS1* and *HS3ST2* represent promising statistical biomarker candidates correlated with alterations in MQC and depression phenotypes. Importantly, all gene prioritization results are derived from computational modeling, including SHAP importance ranking, differential expression analysis, and ROC discriminatory performance. These statistical outputs strongly support their potential value as predictive biomarker signatures for MDD, although they do not yet establish these genes as functionally validated drivers of disease with definitive causal roles in MQC dysfunction or depression progression. The precise biological functions of *DCHS1* and *HS3ST2* in neuropsychiatric disorders and mitochondrial quality modulation remain largely uncharacterized, and current associations should be interpreted cautiously as hypothesis-generating observations rather than confirmed mechanistic conclusions.

*DCHS1* and *HS3ST2* may serve as key biomarkers for MQC and depression. However, the current biological evidence supporting this association remains limited. The role of *DCHS1* in neuropsychiatric disorders or MQC is still unclear, and findings are primarily based on computational predictions. While these results are intriguing, they require cautious interpretation. Further experimental studies, particularly in cellular and animal models, are essential to verify the biological relevance of *DCHS1* and *HS3ST2* and clarify their potential contributions to disease pathogenesis. Given the scarcity of studies linking *DCHS1* and *HS3ST2* to MQC, pathway analyses of these genes were conducted. Computational inference based on GSEA revealed that *DCHS1* and *HS3ST2* are co-enriched in the OXPHOS and cytokine-cytokine receptor interaction pathways. These statistical enrichments suggest potential functional linkages but do not provide biochemical evidence of altered pathway activity or mitochondrial dysfunction. The OXPHOS pathway is central to mitochondrial energy metabolism, and significant evidence indicates OXPHOS impairment in specific brain regions of MDD patients, such as the dlPFC (54). In animal models, corticosterone-induced depressive behavior corresponds with impaired expression of neuronal OXPHOS proteins, which can be corrected through pharmacological treatment (55). Additionally, the gut microbiota regulates hypothalamic activities that influence OXPHOS and the extracellular matrix (56), indicating a direct interaction between mitochondrial energy metabolism and the neuro-endocrine-immune network. The cytokine-cytokine receptor interaction pathway is essential for inflammation. The mitochondrial function protein HKDC1 can inhibit reactive oxygen species (ROS) generation and apoptosis, thus sustaining mitochondrial function while directly repressing the cytokine-cytokine receptor pathway (57). This suggests direct communication between mitochondrial function and inflammatory signaling. Dysfunctional activation of inflammatory pathways (e.g., JAK/STAT, NF-κB) is closely associated with treatment resistance in MDD. High levels of pro-inflammatory cytokines (e.g., IL-6) can trigger hyperactivation of the HPA axis, disrupt neurotransmitter metabolism patterns, and correlate with depression (58). Based on these computational predictions, a hypothesis emerges that *DCHS1* and *HS3ST2* may be statistically associated with MDD pathogenesis, as well as with neuroimmune inflammation and mitochondrial energy metabolic alterations, possibly linked to modulating the threshold of inflammatory signal activation controlled by the MQC system (59). However, this hypothesis necessitates direct biochemical validation through measurements of mitochondrial respiratory chain complex activity, OCR, or ATP generation in future studies.

We are particularly focused on the central role of non-neuronal cells in depression. The present study identified a decrease in the abundance of astrocytes, OPCs, and endothelial cells in MDD patients. Specifically, lower levels of astrocyte markers were observed across multiple brain regions, with reduced densities of labeled astrocytes, suggesting a loss of these cells in individuals with this psychiatric disorder (60). During the progression of MDD, astrocytes mediate neuroinflammation and selectively infiltrate inflammatory areas by crossing the blood-brain barrier (61). This finding indicates that dysregulation of multi-cellular communication among neurons, glia, and vascular cells is associated with the pathogenesis of MDD. Further investigation into the relationship between neuronal abundance, astrocytes, and endothelial cells in MDD holds significant clinical importance. Additionally, ITpr2-mediated calcium homeostasis in oligodendrocytes is essential for myelination and has been implicated in depression-like behaviors in adolescent mice (62). Thus, abnormalities in astrocytes and OPCs are linked to neuroinflammation and cell development, both of which may play key roles in the pathophysiological processes of MDD. Another noteworthy finding was that MQCG activity exhibited significant heterogeneity across cell types and was downregulated in various excitatory and inhibitory neurons, as well as in OPCs—many of which have been shown to be critical players in depression (62–64). This downregulation suggests that their energy metabolism and mitochondrial functions may be under substantial stress or might undergo adaptive or compensatory changes in MDD.

Our research revealed that the two genes, *DCHS1* and *HS3ST2*, exhibited abnormal expression in the Inhib_GRIK1 cells of the PFC of patients with depression. Notably, these cells showed high levels of both genes. There are few reports linking Inhib_GRIK1 cells directly to depression. This cell type was classified into distinct clusters based on the expression of GAD1, GAD2, GRIK1, and ADARB2, but did not express VIP or SST. GAD1 and GAD2 are associated with GABAergic neurons, GRIK1 belongs to the glutamate receptor system, and ADARB2 is related to RNA editing. Among these, the GRIK1 gene (glutamate ionotropic receptor kainate type subunit 1) is primarily associated with neuropsychiatric disorders (65). A notable large-scale postmortem study revealed significantly higher GRIK1 expression levels in the dlPFC of female MDD patients compared to control subjects (66), thereby establishing a direct link between GRIK1 and the pathology of human depression. As an inhibitory neuron, Inhib_GRIK1 is closely related to neural function, particularly neurotransmitter synthesis and neural circuitry. Studies indicate that γ-aminobutyric acid (GABA), the major inhibitory neurotransmitter in the brain, is impaired in MDD (67). This disruption of inhibitory control may hinder the normal regulation of excitatory neurotransmission in the disorder (68). Consequently, MDD is considered a disorder characterized by cortical disinhibition, wherein dysregulation of inhibitory neuronal systems is associated with decreased emotional regulation and increased rumination. Certain subregions of the PFC are thought to be the source of this disinhibition (69). Our data demonstrate that *DCHS1* and *HS3ST2* are differentially expressed in Inhib_GRIK1 cells, and that MQRG activity is reduced in multiple inhibitory subtypes. However, these transcriptional observations do not directly indicate alterations in GABA synthesis, release, receptor function, or synaptic inhibition. Electrophysiological recordings, microdialysis, or synaptic vesicle assays were not performed; therefore, any connections between changes in MQC-related genes and inhibitory neurotransmission remain correlational and serve as hypothesis-generating observations.

We also discovered that the NRG3-ERBB4 ligand-receptor pair exhibited the highest communication probability, as computationally inferred from transcriptomic expression data in both MDD patients and controls, with EX_CUX2 serving as the primary signal source. Notably, this communication probability is derived solely from gene expression-based statistical inference via the CellChat algorithm, rather than from direct functional measurements of pathway activity. These findings are consistent with prior studies indicating that alterations in NRG3-ErbB4 signaling are implicated in a range of psychiatric disorders, including anxiety, depression (70, 71), Hirschsprung’s disease (72), schizophrenia (SCZ) (73), AD (74), Parkinson’s disease (PD) (75), attention deficit/hyperactivity disorder (ADHD) (70), and autism spectrum disorder (ASD) (76). While the direction of our computational predictions aligns with these published observations, we did not perform pharmacological or genetic interventions (e.g., NRG3-ERBB4 agonists/antagonists or gene knockdown) to functionally validate this pathway. EX_CUX2 was not only abundant in MDD but also identified as one of the key cells associated with decreased MQCG activity, potentially suggesting a key “vicious cycle” or “decompensation” mechanism in MDD pathology. We observed an increased relative abundance of EX_CUX2 cells in the MDD group, alongside reduced computationally inferred MQC activity in this subtype. Although this co-occurrence might suggest a possible link between changes in cell abundance and mitochondrial stress, we cannot distinguish whether this increase reflects enhanced proliferation, reduced apoptosis, altered migration, or other mechanisms, as we did not assess cell cycle markers (e.g., Ki67, EdU) or apoptosis assays. It remains unclear whether this change in abundance is a primary event, a compensatory response to network dysfunction, or an epiphenomenon. Therefore, any interpretation of “compensatory proliferation” driving MQC decline remains speculative. The NRG3-ERBB4 pathway may represent a promising candidate target for future research into antidepressant mechanisms; however, functional validation is urgently needed (77). Investigating the NRG3-ErbB4 pathway is expected to yield valuable insights into the mechanisms underlying depression, making it a worthwhile direction for exploration. Nevertheless, confirming these computational predictions through pathway intervention experiments (e.g., NRG3-ERBB4 blockade or activation in animal models, followed by assessments of depressive-like behaviors and MQC function) is critically required.

This study identified *DCHS1* and *HS3ST2* as candidate biomarkers with discriminatory potential for MDD, established through multi-omics integration and machine learning analysis. These genes were statistically associated with disease status and computationally inferred MQC-related transcriptional activity. However, transitioning from a statistical association to determining a causal or regulatory role necessitates further functional validation, such as knockdown or overexpression assays. While our enrichment analyses suggest *DCHS1* and *HS3ST2* may be linked to OXPHOS and cytokine-related pathways, current findings do not establish that these genes directly regulate MQC or MDD pathogenesis. Further experimental studies are needed to clarify their precise roles in neuronal function and mitochondrial homeostasis.

To integrate the multi-omics findings, a conceptual framework linking MQC dysfunction to MDD pathogenesis is proposed (**Figure 1**). At the molecular level, the machine learning model identified *DCHS1* and *HS3ST2* as hub genes. *DCHS1*, which is involved in neuronal migration and polarity, exhibits decreased expression, potentially impairing the structural integrity necessary for mitochondrial dynamics. Conversely, *HS3ST2*, associated with protein aggregation, is upregulated, which may exacerbate proteotoxic stress within mitochondria. At the cellular level, these alterations converge specifically within the Inhib_GRIK1 inhibitory neuron subtype, demonstrating the most significant dysregulation of MQC activity. This cell-type-specific vulnerability disrupts local microcircuits, as indicated by altered ligand-receptor interactions. Notably, the NRG3-ERBB4 signaling pathway, crucial for inhibitory synapse maturation, exhibits the highest communication probability. It is hypothesized that MQC dysfunction in Inhib_GRIK1 cells weakens their inhibitory control over excitatory neurons (e.g., EX_CUX2), potentially leading to a state of cortical hyperexcitability or disinhibition, which is a recognized hallmark of MDD pathophysiology. This multi-layered correlational cascade provides a novel hypothesis-generating model to guide future mechanistic exploration, rather than implying definitive causal pathogenic pathways.

This study presents several inherent limitations. First, all analyses were conducted retrospectively using publicly archived datasets. Heterogeneity among independent cohorts and incomplete clinical metadata (e.g., medication history, PMI) may introduce confounding biases into the results. Although multiple machine learning algorithms were employed to mitigate such interference, adjusting for available clinical covariates and adopting stringent filtering cutoffs, residual confounding effects cannot be entirely eliminated. Additionally, the relatively small sample size of each cohort may compromise sensitivity in detecting low-abundance genes or transcripts with subtle expression shifts. Second, bulk microarray profiling and snRNA-seq differ fundamentally in experimental design and raw data architecture. Cross-platform comparisons via pseudo-bulk transformation only reflect consistent transcriptional trends across technologies; such outcomes should be interpreted as supportive correlational evidence rather than definitive validation. Moreover, mitochondrial transcripts were pre-depleted during the upstream processing of the snRNA-seq dataset, which precludes direct quantification and evaluation of mitochondrial gene expression at the single-nucleus level. Third, the CRS rat model cannot fully recapitulate the comprehensive spectrum of human MDD pathophysiology. The analysis exclusively focused on the PFC, limiting the profiling of molecular alterations occurring in other brain regions implicated in depression. Fourth, functional validation in the current work remains incomplete from multiple perspectives: (a) No proliferation (Ki67, EdU) or apoptosis assays were conducted, leaving the precise mechanisms underlying altered abundances of cell subtypes such as EX_CUX2 uncharacterized; (b) Electrophysiological recordings and synaptic functional assessments are absent, leaving the causal linkage between transcriptomic shifts in MQC and impaired GABAergic inhibitory function unconfirmed; (c) A complete causal chain encompassing molecular genes, cellular phenotypes, intercellular circuits, and behavioral phenotypes has not been established. Consequently, all findings presented herein are hypothesis-generating observations that necessitate rigorous follow-up experimental validation. Fifth, GSEA and cell-cell communication inference are purely computational outputs derived from transcriptomic profiles, which do not equate to actual metabolic pathway activity, mitochondrial respiratory capacity, or functional ligand-receptor signaling at the protein level. The regulatory role of cytokine signaling pathways was not validated through protein quantification. Additionally, while the association between *HS3ST2* and tau protein aggregation was originally reported in AD research, whether this regulatory relationship is generalizable to MDD remains to be clarified. Future investigations should encompass larger human MDD patient cohorts and integrate spatial transcriptomics alongside systematic functional assays, including gene editing, electrophysiology, and Seahorse extracellular flux metabolic profiling. Additional experimental work is warranted to measure tau protein post-translational modifications and pro-inflammatory cytokine protein levels in MDD models and clinical samples. Targeted intervention of the NRG3-ERBB4 signaling axis will also aid in dissecting its potential interplay with MQC dysfunction underlying depressive pathology.

## 5 Conclusions

In conclusion, this study integrated public transcriptomic datasets, snRNA-seq data, machine learning algorithms, and mRNA verification from the CRS rat model to identify *DCHS1* and *HS3ST2* as candidate biomarkers associated with MQC-related transcriptional alterations in MDD. MQRG activity exhibited significant cell-type heterogeneity and was diminished in multiple PFC cell populations, including various neuronal and glial subtypes. These findings provide preliminary, correlational insights into MQC-associated transcriptional dysregulation in MDD. However, as mRNA-level verification in the CRS rat model solely reflects transcriptional changes and cannot distinguish direct regulatory effects from compensatory alterations, functional studies are still required to ascertain whether *DCHS1* and *HS3ST2* directly regulate MQC or contribute causally to MDD pathogenesis.

## Data Availability

Publicly available datasets were analyzed in this study. This data can be found here: https://www.ncbi.nlm.nih.gov/geo/query/acc.cgi?acc=GSE92538 https://www.ncbi.nlm.nih.gov/geo/query/acc.cgi?acc=GSE144136 https://www.ncbi.nlm.nih.gov/geo/query/acc.cgi?acc=GSE54570 https://doi.org/10.5281/zenodo.21447176.
