# Supplementary material for: Integrative multi-omics and machine learning analysis identifies candidate biomarkers associated with mitochondrial quality control in major depressive disorder
Source: Front Psychiatry. 2026 Aug 12;17:1828806. doi: 10.3389/fpsyt.2026.1828806 (PMC13506908; doi:10.3389/fpsyt.2026.1828806)
Supplement: Supplementary file 1 [file DataSheet1.zip › Supplementary Materials/Supplementary_Material FIGURE1-4.docx]

Supplementary Material

**Figure S1**

**Figure S1.** Line plot of feature selection using the RFE algorithm (A) LR；(B) DT；(C) SVM；(D) RF；(E) KNN；(F) BT；(G) LDA；(H) NNET；(I) NB ( Line plots display the accuracy and Kappa values from 10-fold cross-validation for different numbers of features selected by RFE using various algorithms. The red dashed line indicates the optimal number of features for each model.)

***Figure S2***

**Figure S2.** (A) Violin plot of pre-filtering quality control metrics; (B) Violin plot of post-filtering quality control metrics; (C) Selection of highly variable genes; (D) PCA scree plot; (E) Elbow plot visualizing principal components; (F) The distribution of computationally inferred MQRGs enrichment scores in single cells; (G) Intergroup differential analysis of computationally inferred MQRGs enrichment scores between MDD and control groups.

***Figure S3***

**Figure S3.** Expression differences of DCHS1 and HS3ST2 across cell types. Dot-boxplot showing the expression levels of DCHS1 (left) and HS3ST2 (right) in each annotated cell type between MDD and control groups. The x-axis represents different cell types, and the y-axis shows the normalized expression level. Each dot represents an individual cell. Statistical significance was assessed using the Wilcoxon rank-sum test. ns, not significant; **P* < 0.05; unmarked comparisons indicate *P* < 0.05.

***Figure S4***

**Figure S4.** (A) Line plot of principal components; (B) PCA scree plot; (C) UMAP plot of key cell subpopulations; (D) Heatmap of marker genes for subpopulation annotation.
